# Supplementary material for: Persistence of the Immune Response to an Intramuscular Bivalent (GI.1/GII.4) Norovirus Vaccine in Adults
Source: Vaccines (Basel). 2025 Jan 17;13(1):82. doi: 10.3390/vaccines13010082 (PMC11768790; doi:10.3390/vaccines13010082)
Supplement: Supplementary file 1 [file vaccines-13-00082-s001.zip › vaccines-3383782-supplementary.pdf]

SUPPLEMENTARY MATERIAL

Persistence of the Immune Response to an Intramuscular Bivalent (GI.1/GII.4) Norovirus Vaccine in Adults

Geert Leroux-Roels [1], Robert L. Atmar [2], Jakob P. Cramer [3,4], Ian Escudero [3,5] and Astrid Borkowski [3,6,*]

[1] Center for Vaccinology (CEVAC), Ghent University and University Hospital, 9000 Ghent, Belgium; geert.lerouxroels@ugent.be

[2] Departments of Medicine and Molecular Virology and Microbiology, Baylor College of Medicine, Houston, TX 77030, USA; ratmar@bcm.edu

[3] Clinical Development, Takeda Pharmaceuticals International AG, Farman Strasse 11, Opfikon, 8152 Zurich, Switzerland; jakob.cramer@cepi.net (J.P.C.); ian.escudero@takeda.com (I.E.)

[4] Clinical Development, Coalition for Epidemic Preparedness Innovations (CEPI), Gibbs Building, 215 Euston Road, London NW1 2BE, UK

[5] Clinical Development, Takeda Vaccines, 75 Sidney St., Cambridge, MA 02139, USA

[6] HilleVax AG, Boulevard Lilienthal 42, Opfikon, 8152 Zurich, Switzerland

* Correspondence: aborkowski@hillevax.com

Supplementary Table S1

Demographic characteristics in the FAS, PPS and ASC subset

**Category**		**FAS N=528**	**PPS N=525**	**CMI Subset N=234**
			Mean (SD)	
Period from 1st Dose to Last Visit (years)		4.9 (0.4)	4.9 (0.4)	4.9 (0.4)
Age (years):	Overall	53.6 (18.8)	53.7 (18.8)	54.9 (18.3)
			n (%)	
Age range: (years)	18–64	405 (76.7)	402 (76.6)	175 (74.8)
	65–84	86 (16.3)	86 (16.4)	46 (19.7)
	≥85	37 (7.0)	37 (7.0)	13 (5.6)
Female		324 (61.4)	322 (61.3)	146 (62.4)
Ethnicity:	Not Reported	345 (65.3)	342 (65.1)	158 (67.5)
	Not Hispanic or Latino	168 (31.8)	168 (32.0)	69 (29.5)
	Hispanic or Latino	15 (2.8)	15 (2.9)	7 (3.0)
Race:	White	510 (96.6)	507 (96.6)	230 (98.3)
	Black or African American	12 (2.3)	12 (2.3)	3 (1.3)
	Asian	4 (0.8)	4 (0.8)	0 (-)
	Native Hawaiian or Other Pacific Islander	1 (0.2)	1 (0.2)	1 (0.4)
	Multiracial (a)	1 (0.2)	1 (0.2)	0 (-)

Demographic characteristics were taken from when the participant signed the informed consent form in the initial trial. Percentage of participants was calculated as 100×n/N, where n=number of participants with the characteristic, and N=number of participants. Note, ethnicity was not collected for participants from NOR-107. (a) Participants checked more than one race option on the case report form. CMI=cell-mediated immunity. FAS=full-analysis set. PPS=per-protocol set.

Supplementary Figure S1

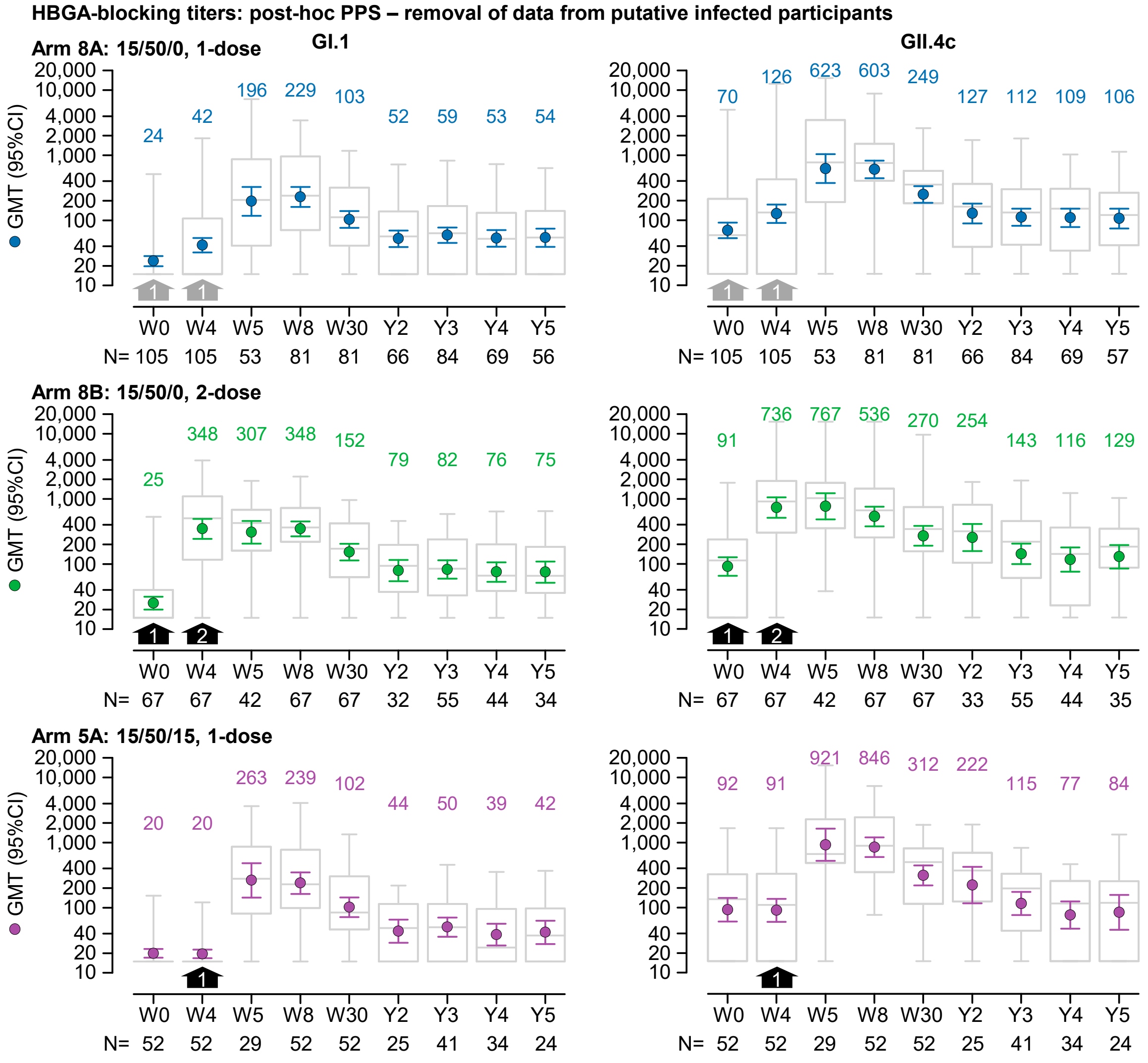

GI.1-specific and GII.4c-specific HBGA-blocking geometric mean titers (GMTs; left and right graphs, respectively) over a 5-year period from prior to dosing (Week 0; W0) in the preceding trials to the end of the present follow-up study (Year 5, Y5) in the per-protocol set (PPS) without data from participants putatively infected with norovirus (with either a GI.1-like or GII.4-like genotype and identified by ≥4-fold increase in antibody titer at a subsequent visit during the follow-up period). Three dosage regimens are shown: (i) 1 dose of 15/50/0 (either at W0 or W4; grey arrows); (ii) 2 doses of 15/50 (at W0 and W4; black arrows); and 1 dose of 15/50/15 at W4 (black arrows). GMTs and 95% confidence intervals are represented by colored round symbols (and value above the plot) and error bars, respectively. Medians, 25 and 75 percentiles, minimum and maximum values are represented by grey box and whisker plots.

Supplementary Figure S2

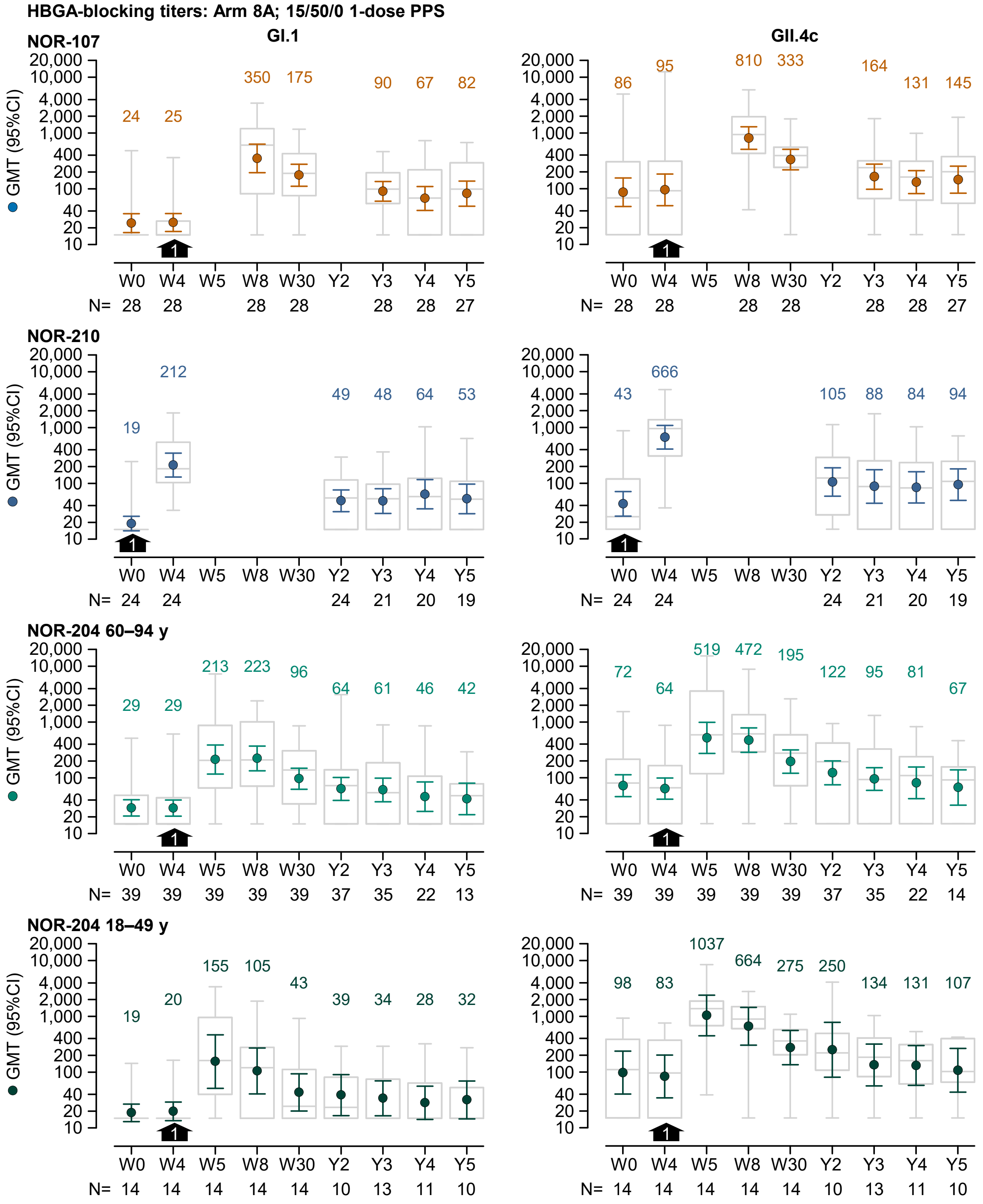

GI.1-specific and GII.4c-specific histo-blood group antigen (HBGA)-blocking geometric mean titers (GMTs; left and right graphs, respectively) over a 5-year period from prior to dosing (Week 0; W0) in the preceding trials (NOR-107, NOR-210, and NOR-204) to the end of the present follow-up study (Year 5, Y5), for recipients of a single dose of 15/50/0 (Arm 8A) in the per-protocol set (PPS). The vaccine was administered at either at W0 or W4 (black arrows). GMTs and 95% confidence intervals are represented by colored round symbols (and value above the plot) and error bars, respectively. Medians, 25 and 75 percentiles, minimum and maximum values are represented by grey box and whisker plots.

Supplementary Figure S3

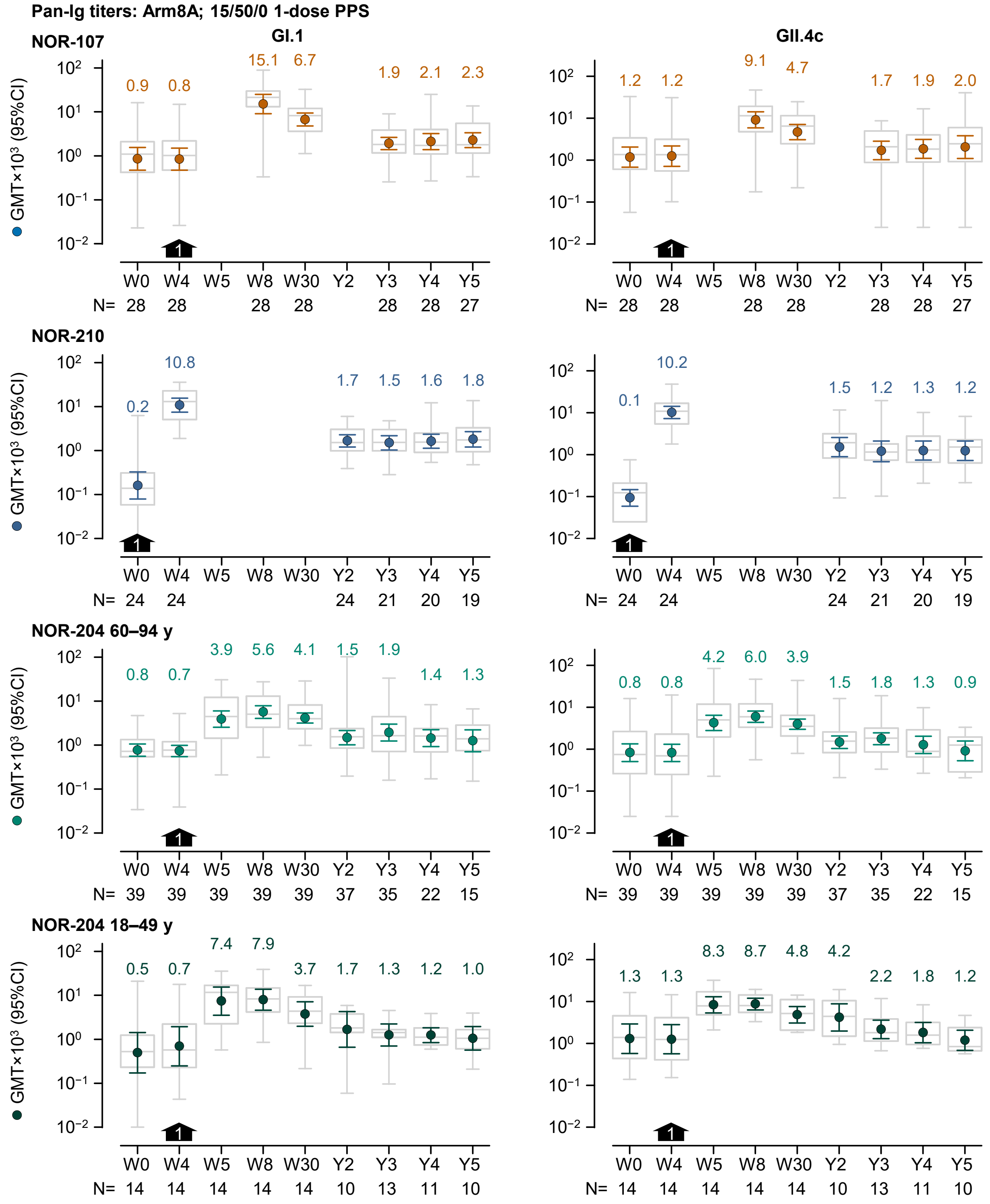

GI.1-specific and GII.4c-specific pan-Ig geometric mean titers (GMTs; left and right graphs, respectively) over a 5-year period from prior to dosing (Week 0; W0) in the preceding trials (NOR-107, NOR-210, and NOR-204) to the end of the present follow-up study (Year 5, Y5), for recipients of a single dose of 15/50/0 (Arm 8A) in the per-protocol set (PPS). The vaccine was administered at either at W0 or W4 (black arrows). GMTs and 95% confidence intervals are represented by colored round symbols (and value above the plot) and error bars, respectively. Medians, 25 and 75 percentiles, minimum and maximum values are represented by grey box and whisker plots. Note, the GMTs in the figure are expressed in a scientific notation that includes a 10^3 multiplier.

Supplementary Figure S4

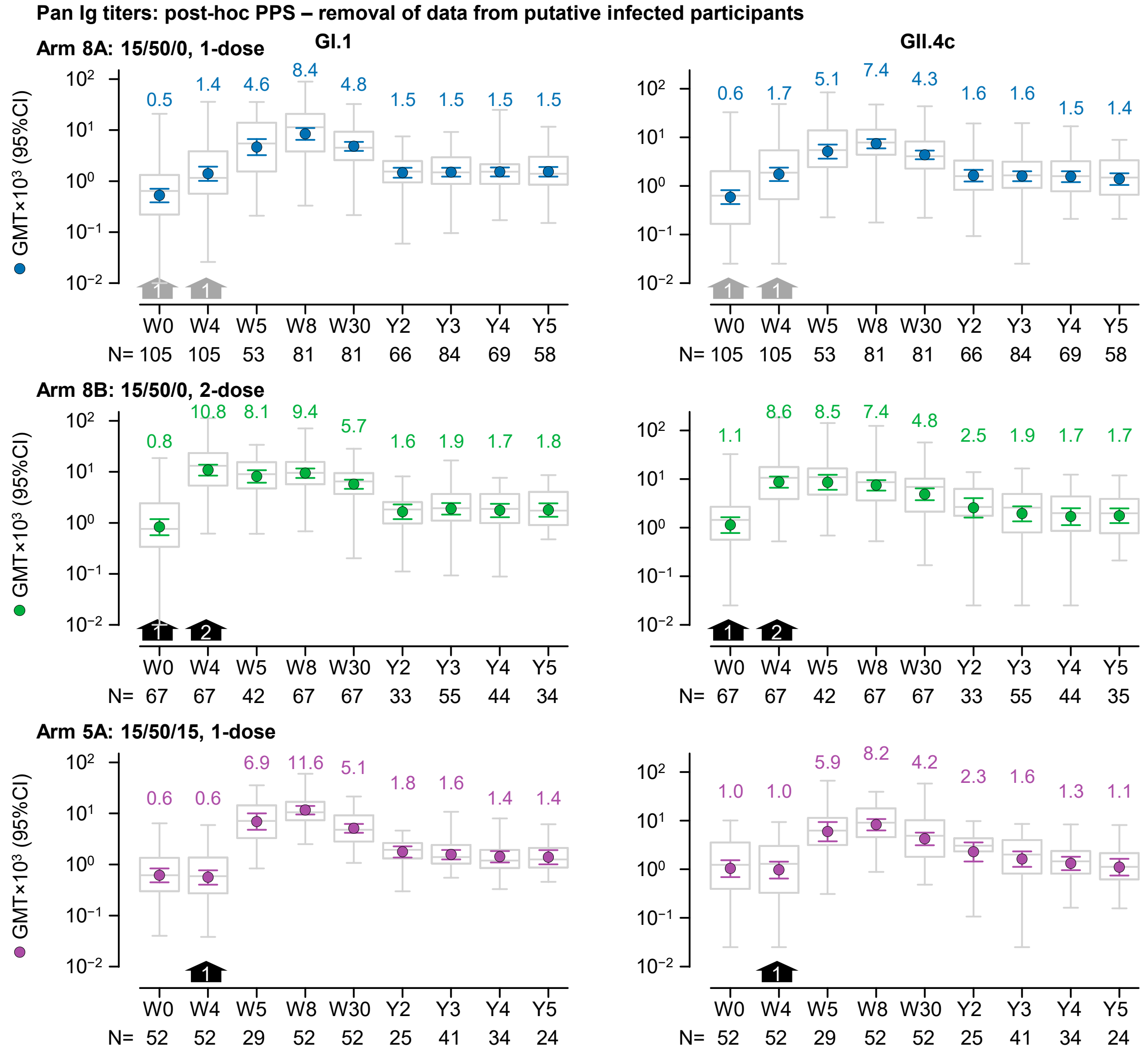

GI.1-specific and GII.4c-specific pan-Ig geometric mean titers (GMTs; left and right graphs, respectively) over a 5-year period from prior to dosing (Week 0; W0) in the preceding trials (NOR-107, NOR-210, and NOR-204) to the end of the present follow-up study (Year 5, Y5), for recipients of a single dose of 15/50/0 (Arm 8A) in the per-protocol set (PPS) without data from participants putatively infected with norovirus (with either a GI.1-like or GII.4-like genotype and identified by ≥4-fold increase in antibody titer at a subsequent visit during the follow-up period). The vaccine was administered at either at W0 or W4 (black arrows). GMTs and 95% confidence intervals are represented by colored round symbols (and value above the plot) and error bars, respectively. Medians, 25 and 75 percentiles, minimum and maximum values are represented by grey box and whisker plots. Note, the GMTs in the figure are expressed in a scientific notation that includes a 10^3 multiplier.
